# Dreams, Sleep, and Psychotropic Drugs

**DOI:** 10.3389/fneur.2020.507495

**Published:** 2020-11-05

**Authors:** Alain Nicolas, Perrine M. Ruby

**Affiliations:** Lyon Neuroscience Research Center, CNRS UMR 5292 - INSERM U1028 - Lyon 1 University, Lyon, France

**Keywords:** sleep, dream recall frequency, dream content, antidepressant, antipsychotic, anxiolytic, dreaming

## Abstract

Over the past 60 years, the impact of psychotropic drugs on dream recall and content has been scarcely explored. A review of the few existing experimental results on the topic leads us to the following conclusions. For antidepressant drugs, in the great majority, they reduce dream recall frequency (DRF), and the improvement of depressive symptoms is associated with an increase of positive emotion in dream content. For sedative psychotropic drugs, their improvement of sleep quality is associated with a reduction of DRF, but the effect on dream content is less clear. Few occurrences of nightmare frequency increase have been reported, with intake of molecules disturbing sleep or with the withdrawal of some psychotropic drugs. Importantly, the impact of psychotropic drugs on rapid eye movement (REM) sleep does not explain DRF modulations. The reduction of intra-sleep awakenings seems to be the parameter explaining best the modulation of DRF by psychotropic drugs. Indeed, molecules that improve sleep continuity by reducing intra-sleep awakenings also reduce the frequency of dream recall, which is coherent with the “arousal-retrieval model” stating that nighttime awakenings enable dreams to be encoded into long-term memory and therefore facilitate dream recall. DRF is nonetheless influenced by several other factors (e.g., interest in dreams, the method of awakening, and personality traits), which may explain a large part of the variability of results observed and cited in this article.

## Key Points

- Most antidepressants reduce dream recall frequency. Dream content tends to improve in parallel to improvements in symptoms of depression.
- Sedative psychotropic drugs reduce dream recall frequency by improving sleep quality, but their impact on dream content is not well-known.
- The influence of psychotropic drugs on REM sleep does not modify their impact on dream recall frequency.
- Decrease of intra-sleep awakenings due to taking psychotropic drugs is the variable, which most correlates with dream recall frequency.

## Introduction

Dreaming is an experience shared by everyone; very few people report having never dreamt [e.g., (1, 2)]. This mysterious and ubiquitous phenomenon is however difficult to investigate with an experimental approach, since researchers can access dreams only indirectly and *a posteriori* via dream reports possibly truncated by partial memory and transformed by the awake mind (3).

As a consequence, the definition of dreaming is still under debate (4, 5). The following phenomenological one is nonetheless shared and used by numerous dream researchers [e.g., (2, 6, 7)]: “Dreaming is a spontaneous phenomenon during sleep which is a true phenomenal experience, i.e., it feels as an experience of the waking life (loss of reality testing). It is composed of “virtual” sensory perceptions and of emotions and it can evoke various and complex representations of the setting, characters, objects and circumstances. The dreamer is therefore both the unaware creator of the dream and its conscious observer and often actor” (7). In other words, most of the time dreams are experienced as real, and the dreamer is unaware of being asleep except in the peculiar case of lucid dreaming (8). Importantly, oneiric representations can be bizarre. After awakening, dreams may be recalled due to the recollection—often fleeting—of the dream memory.

Regarding the link between dreaming and neurophysiology, 60 years of debates has been necessary to get rid of the rapid eye movement (REM) sleep hypothesis of dreaming, and it is now acknowledged by a majority of dream researchers, be they neuroscientist or not, that REM sleep is neither necessary nor sufficient to get a dream report and that dream reports can be obtained at the awakening of any sleep stage (2, 7, 9–12). It is also now clear that the complexity and story-like characteristics of dream reports are related to sleep stages but also, and even more so, to circadian rhythms leading to more vivid and complex dreams in the morning than in the early night [e.g., (13–15)]. Recent developments have provided strong experimental arguments in favor of the arousal-retrieval model (16), proposing that awakenings when dream content is still in short-term memory are necessary to encode dreams into long-term memory (17–23).

Several studies have investigated the effects of psychotropic drugs on the structure of sleep (24). However, studies testing the impact of these substances on reported dreams are rarer. The objective of this review is to present a synthesis of these two bodies of research.

## Effects of Antidepressants on Dream Recall

Although sleep disorders and complaints about dreams and nightmares are common in depressed individuals (whether they are under treatment or not) (25), only few studies have tested at the experimental level the effects of antidepressants on dream recall [see (26), for a review proposing a table with the antidepressant tested and their effect on dreams in depressed and healthy individuals]. We favored data obtained from healthy subjects when such data exist, in order to avoid the confounding factor of depression. Indeed, depressed patients often report less dreams than healthy participants (27–29), and the tone of their dreams is often more negative (26, 30).

The effects of antidepressants on the structure of sleep are rather well-documented. Tricyclic antidepressants tend to improve the quality and duration of sleep (24, 31), with some being often used to treat chronic insomnia (32). In contrast, most selective serotonin reuptake inhibitor (SSRI) antidepressants reduce the duration of sleep, increase its fragmentation, and present an insomniac profile (33). The great majority of these substances have a significant impact on REM sleep, by extending its latency period and reducing its duration (34, 35).

### Monoamine Oxidase Inhibitor Antidepressants

The only monoamine oxidase inhibitor antidepressant (MAOI) for which we have any data is phenelzine. A further limitation is the fact that data are only available for depressed subjects. Landolt et al. demonstrated that depressed patients who were responsive to *phenelzine* manifest reduction of dream recall, but not those who were unresponsive, although suppression of REM sleep was equivalent in both groups (36). A positive impact of phenelzine treatment on the frequency of nightmares in patients suffering from post-traumatic stress disorder (PTSD) has also been reported (37). Finally, withdrawal, if sudden, seems to provoke a rebound of dream recall essentially comprising nightmares in both patients (38, 39) and healthy subjects (40).

### Tricyclic Antidepressants

In healthy subjects, *imipramine* reduced the frequency of dream recall in at least two studies (41, 42). This effect stands in depressed patients, even if less significant. Furthermore, improvement of dream content has been observed in parallel to improved mood (43) but not in all studies (44). As with MAOIs, withdrawal generally engenders an increase in nightmares and negative impressions during dreaming (45).

*Clomipramine* does not seem to modify much dream activity (46). This is corroborated by Oudiette et al. (47), who found no major effect on dreams in healthy subjects taking clomipramine, other than increased “strangeness” in sleep stage 2 (N2) dream reports and increased length of sleep stage 1 (N1) dream reports. Interestingly, the molecule would be responsible for an increased risk of REM sleep behavior disorder (RBD) according to Hoque and Chesson (48). RBD is a loss of muscle atonia during REM sleep leading to a playing out of the dreamt actions and possibly causing injuries to patients and their entourage (49).

*Amitriptyline* did not cause a change in dream activity in healthy volunteers, even after prolonged administration, in the few studies that used a reliable method for collecting dream-related data (46, 50). Similar results were found in depressed patients (46). With the use of an unstructured questionnaire, perturbation in dreams was detected after withdrawal, but most of the patients also demonstrated signs of relapse into depression (51).

*Trimipramine* is a different kind of tricyclic antidepressant, as it is the only one in its category that does not provoke any significant alteration in REM sleep. A treatment lasting 4 weeks provoked a reduction in dream recall and a significant “improvement” in dream content in depressed patients, in conjunction with remission of symptoms of depression (52, 53).

Note that in several studies that have used a sample of patients taking tricyclic or neuroleptic drugs in one single (often high) dose before bedtime, an increase in frightening dreams was consistently reported, but this effect was not observed when the dose was divided and administered over the course of the day (54, 55).

### Selective Serotonin Reuptake Inhibitor Antidepressants

*Fluoxetine* is one of the rare antidepressants that increase dream recall frequency (44, 56). This molecule tends to increase also nightmare recall (57) and the intensity of dreams reported (58, 59). When such intensification occurs in a negative context, it is nonetheless associated with improvements in symptoms of depression (60). Fluoxetine has also been incriminated in provoking episodes of RBD (61).

*Paroxetine* reduces the frequency of dream recall but increases intensity of dream content (memorability, visual intensity, quantity of sound, emotional intensity, and significance) both during treatment and also when suddenly withdrawn (62). It may also be responsible for activating RBD (63), while it would have a fairly positive effect on PTSD nightmares (64).

The action of *fluvoxamine* is very similar to that of paroxetine (62) with a reduction in dream recall and modification of dream intensity, but withdrawal results in an increase in both the strangeness of dreams and in the number of words used to report them.

*Escitalopram* was shown to increase dream recall frequency, in parallel with improvement of symptoms in depressed patients (65). Improvement of dream content was also reported: they are less complex and more intense at the emotional level. *Citalopram* also increased dream recall in a group of patients treated for obsessive compulsive disorder (66).

### Serotonin–Noradrenaline Reuptake Inhibitor Antidepressants

*Venlafaxine* and its metabolite, *desvenlafaxine*, also tend to modify the dream content, with a large increase of “abnormal dreams” reported in desvenlafaxine withdrawal in depressed patients (67). With venlafaxine, an emergence of particularly realistic nightmares is observed (68) as well as episodes of RBD (69).

*Duloxetine* demonstrates effectiveness in reducing PTSD nightmares (70) and has a similar effect on sleep as venlafaxine (71): reduced length of REM sleep, fragmented sleep, and increase in periodic limb movement. For *milnacipran*, no increase in dream recall was observed (72).

### Other Antidepressants

*Mianserin* is an antidepressant with a well-known sedative effect (24). It induces a reduction in dream recall according to Besançon et al. (56). *Mirtazapine*, which has similar properties, may be useful for treating PTSD nightmares (73) but was also reported to induce nightmares (74). Note that REM sleep is partially suppressed by mianserin intake whereas mirtazapine has nearly no impact on REM sleep (75).

*Trazodone* inhibits recapture of serotonin and blocks 5HT_2A_ and 5HT_2C_ receptors (76, 77). It is widely used throughout the world as a treatment for insomnia. A withdrawal syndrome from trazodone induces a nightmare increase (78). During treatment, this molecule reduces nightmare in patients suffering from depression (79), PTSD (80), or cancer (81). *Nefazodone* (similar to trazodone) also inhibits recapture of norepinephrine (82, 83). It has little effect on REM sleep in depressed patients (84) and reduces dream recall frequency (44). Regarding dream content, five of the 18 parameters investigated showed a reduction (intensity, number of other people, presence of the body, sexual content, and number of scenes). Note that some authors observed an increase in REM sleep in healthy subjects taking nefazodone (85). This molecule seems to be also efficient for treating nightmares in PTSD patients (86).

*Bupropion* is a low-strength dopamine–norepinephrine reuptake inhibitor, and its effect on REM sleep is debated: some argue no effect (87), and others an increase (88). Often used as an adjuvant to SSRIs because it thwarts some of their side effects, it induces very few episodes of RBD (89) and increases dream recall frequency (90, 91). Regarding nightmares, only Balon (92) reported an increase in nightmare frequency in patients treated with bupropion.

Depressed patients taking *tianeptine* [which increases serotonin reuptake; (93, 94)] report increased insomnia complaints and nightmares (95). This molecule does not seem to significantly impact REM sleep (96).

*Agomelatine* is one of the more recent antidepressants on the market. It has an original profile: mainly melatoninergic agonist (MT1 and MT2) but also lightly 5HT_2C_ antagonist (97, 98). It does not influence REM sleep in depressed patients (99), nor in older healthy subjects (100). Regarding dreaming, a significant improvement in PTSD nightmares has been reported in one patient (101).

In summary, antidepressants generally tend to reduce dream recall frequency and to modify dream content, usually positively, in correlation with clinical improvements observed in patients. Most authors suggest that the reduction in dream recall frequency is mainly due to the inhibiting effect of these molecules on REM sleep. However, some antidepressants such as trimipramine do not reduce REM sleep but do reduce dream recall (52), and some such as clomipramine diminish REM sleep duration but have no effect on dream recall frequency (46), while others such as fluoxetine strongly inhibit REM sleep and increase dream recall frequency (24).

One may wonder which mechanism is at work since very different molecules with different modes of action have a relatively uniform impact on dream activity. What all these molecules have in common is the fact that they are antidepressants, and most of them improve the subjective perception of sleep [e.g., (56, 102)]. This may account for the reduction in dream recall frequency and improved dream content tone. The “arousal-retrieval” model (16) proposing that intra-sleep awakenings are a key mechanism in the modulation of dream recall [supported by several experimental results; e.g., (19, 20)] support this hypothesis. In this framework, the reduction of intra-sleep awakenings explains the reduction of dream recall frequency engendered by antidepressants.

## Effects of Antipsychotic Drugs on Dream Recall

First- or second-generation antipsychotic drugs are generally sedative and improve the continuity of sleep without significantly altering the internal structure of sleep (103).

The effect of *chlorpromazine* (first antipsychotic on the market) on dreams recalled both after spontaneous awakenings in the morning and after nighttime awakenings in REM sleep showed that the length of nighttime dreams reported becomes quickly comparable with that obtained in the morning once the individual is in remission, while it is shorter when the individual is in the acute phase (104).

In healthy subjects, *sulpiride* does not significantly modify the structure of sleep. Dreams recalled in the morning are less clear and more emotionally neutral than in a control group. Awakenings in REM sleep result in shorter dream reports, less rich in aggressive or sexual content (105), which corroborates the findings of Karmer et al. (106) in schizophrenic patients treated with first-generation antipsychotics.

Lusignan et al. (107) focused on the characteristics of schizophrenic patients' dream reports, treated in chronic phase of the disorder using *second-generation antipsychotic drugs* (olanzapine, clozapine, quetiapine, or risperidone). Data obtained from questionnaires demonstrated that, under treatment, the group of schizophrenic patients reported more nightmares than healthy subjects, but no other between groups differences were observed for other parameters (number of dream recalls, recurrent dreams, and frequency of specific emotions). During nighttime awakenings in REM sleep, patients reported shorter dreams, but that aside, the parameters of dreams reported were comparable between the two groups, except for the presence of strangers in dreams, more frequently reported by patients.

Finally, a positive effect of *olanzapine* (108), *risperidone* (109), and *aripiprazole* (110) was observed on PTSD nightmares.

## Effects of Anxiolytic and Hypnotic Drugs on Dream Recall

Although different in terms of their pharmacokinetics, benzodiazepines (which represent most of the anxiolytic and hypnotic drugs) are a fairly homogenous group of molecules. They all have sedative, anticonvulsant, muscle relaxing, and probably amnesic effects. Their impact is significant on the structure of sleep with, in particular, a drastic reduction in slow-wave sleep (in favor of N2) but only a low impact on REM sleep (111). They moreover reduce the number of intra-sleep awakenings, essentially in the initial 3–6 weeks of treatment.

*Clonazepam* has probably been the most studied molecule in the field of sleep medicine, due to its numerous potential applications. It is notably often used for PTSD, even if the only study that explored the effect of clonazepam on this disorder reported inconclusive results (112).

Anesthetists have also explored the effects of various benzodiazepines in their field of expertise and, doing so, observed the recall of dreams at postanesthesia awakenings. When associated with ketamine or fentanyl, *diazepam* seems to reduce the number of dreams reported (113). *Midazolam* also reduces dream recall as compared with propofol (114). Finally, taking *quazepam* or *brotizolam* the night before a surgery reduces the number of dreams recalled while increasing the duration and quality of sleep (115).

According to the most well-controlled studies, most of these molecules seem to have little effect on dream recall frequency (116), particularly *nitrazepam* (117), and *flunitrazepam* (118). Regarding dream content, there is a tendency for an increased positive tone in insomniacs under treatment (116). However, in more clinical studies, a reduction in dream recall frequency is observed under *lormetazepam* (119) and *oxazepam* and *midazolam* (120) and a reduction of “anxious dreams” under *diazepam* and *clorazepate dipotassium* (121); and under *oxazepam* and *midazolam*, patients only remember pleasant dreams (120). Similarly, an improvement of “unpleasant dreams” is observed with *triazolam* and *nitrazepam* (122) associated with improvement of sleep quality.

*Eszopiclone*, a hypnotic drug derived from benzodiazepines, seems to stimulate the emergence of “abnormal dreams” upon withdrawal in a small number of older patients (123).

## Effect of Mood Stabilizers on Dream Recall

To our knowledge, no studies directly explored parameters of dream activity for classic mood stabilizers, which are lithium, sodium valproate, valpromide, lamotrigine, carbamazepine, and oxcarbazepine.

In a geriatric sample, it has been reported that *lamotrigine* engenders an increase in dream recall frequency associated with a reduction in sleep duration in approximately 20% of patients, but this assessment is marginal (124).

*Topiramate* and *gabapentin*, which are second- and third-line mood stabilizers, are also cited among therapeutic alternatives that can be used in the case of PTSD (125, 126). They induce a significant reduction of nightmares and improve sleep quality.

## Conclusion

If we want to extract a general image of the impact of psychotropic drugs on dream recall given the available data, the short story is that the overriding majority of molecules engender a reduction in dream recall frequency once they improve the quality of sleep, or the concurrent psychiatric symptoms (Table 1). The few exacerbations of nightmares are caused by some molecules that perturb sleep and also by withdrawal from certain psychotropic drugs.

**Table 1:** Synthesis of the effects of psychotropic drugs on sleep and dreams according to the available data.

**Psychotropic drug category**	**Drug name**	**Sleep continuity**	**REM sleep**	**DRF**	**Nightmare**	**Dream content**
MAOI antidepressant	Phenelzine		–	–	–	
Tricyclic antidepressant	Imipramine	–	–	–		+ positivity
	Clomipramine	–	–	=		+ bizarreness
	Amitriptyline	+	–	=		
	Trimipramine	+	=–	–		+ positivity
SSRI antidepressant	Fluoxetine	=–	–	+	+	+ intensity
	Paroxetine	=–	–	–		+ intensity
	Fluvoxamine			–		+ intensity
	Escitalopram	=–	–	+		+ positivity
IRS-NA antidepressant	Duloxetine	–	–		– in PTSD	
	Milnacipran			=		
Other antidepressants	Mianserin	+	–	–		
	Trazodone	+			–	
	Nefazodone		=	–		– intensity
	Bupropion	+	= +	+	= +	
	Tianeptine		=		+	
Antipsychotic drugs	Chlorpromazine	+				+ length
	Sulpiride	=				– clarity + neutrality
	Second-generation antipsychotic^*^	+		=	+	=
Anxiolytic & hypnotic drugs	Benzodiazepines^..^	+		=–		+ positivity
Mood stabilizers^∧^	Lamotrigine	– sleep duration		+		
	Second- and third-line mood stabilizers”	+			–	

*The review of Wichniak et al. (24) has been used to report the effects of antidepressant drugs on sleep continuity and REM sleep*.

*REM sleep, rapid eye movement sleep; DRF, dream recall frequency; +, increase; –, decrease; =, no modification; MAOI, monoamine oxidase inhibitor antidepressants; SSRI, selective serotonin reuptake inhibitor; IRS-NA, serotonin–noradrenaline reuptake inhibitor*;

* *olanzapine, clozapine, quetiapine, or risperidone*;

.. *midazolam, diazepam, quazepam, brotizolam, nitrazepam, flunitrazepam, lormetazepam, oxazepam, midazolam, and triazolam*;

∧ *lithium, sodium valproate, valpromide, lamotrigine, carbamazepine, and oxcarbazepine; “topiramate and gabapentin; PTSD, post-traumatic stress disorder*.

This makes sense in the framework of the “arousal-retrieval” model (16), stating that nighttime awakenings enable dreams to be encoded into long-term memory and therefore facilitate dream recall. It is thus coherent that molecules that improve sleep continuity by reducing intra-sleep awakenings consequently reduce dream recall frequency. Dream recall frequency is however under the control of several factors [e.g., (17); for a review, (2)], which certainly explain a large part of the variability of the results cited in this article. Some further studies allowing the direct correlation between dream recall and sleep variables in patients off and on psychotropic drugs are nonetheless necessary to confirm the general conclusion suggested by this review.

Regarding dream content, an often reported observation in depressed patients is a concomitant improvement of symptoms (improved mood during wake) and dream content (more positive dreams) after antidepressant intake. This observation fits well with both the continuity hypothesis of wake and dreaming cognition (127) and with the hypothesis of dreaming being involved in emotional regulation [e.g., (128)]. However, these correlational results cannot establish which and if an improvement impacts the other (improved mood causing improved dreams or the other way around), and further studies are needed to progress on this point.

## Author Contributions

AN and PR wrote the article. Both authors contributed to the article and approved the submitted version.

## Conflict of Interest

The authors declare that the research was conducted in the absence of any commercial or financial relationships that could be construed as a potential conflict of interest.
